# Emerging role and therapeutic implication of mTOR signalling in intervertebral disc degeneration

**DOI:** 10.1111/cpr.13338

**Published:** 2022-10-03

**Authors:** Hai‐Wei Chen, Jian‐Wei Zhou, Guang‐Zhi Zhang, Zhang‐Bin Luo, Lei Li, Xue‐Wen Kang

**Affiliations:** ^1^ Department of Orthopaedics Lanzhou University Second Hospital Lanzhou Gansu People's Republic of China; ^2^ The Second Clinical Medical College Lanzhou University Lanzhou Gansu People's Republic of China; ^3^ Key Laboratory of Orthopaedics Disease of Gansu Province Lanzhou University Second Hospital Lanzhou Gansu Province People's Republic of China

## Abstract

Intervertebral disc degeneration (IDD), an important cause of chronic low back pain (LBP), is considered the pathological basis for various spinal degenerative diseases. A series of factors, including inflammatory response, oxidative stress, autophagy, abnormal mechanical stress, nutritional deficiency, and genetics, lead to reduced extracellular matrix (ECM) synthesis by intervertebral disc (IVD) cells and accelerate IDD progression. Mammalian target of rapamycin (mTOR) is an evolutionarily conserved serine/threonine kinase that plays a vital role in diverse degenerative diseases. Recent studies have shown that mTOR signalling is involved in the regulation of autophagy, oxidative stress, inflammatory responses, ECM homeostasis, cellular senescence, and apoptosis in IVD cells. Accordingly, we reviewed the mechanism of mTOR signalling in the pathogenesis of IDD to provide innovative ideas for future research and IDD treatment.

## Graphical abstract

Abnormal autophagy, oxidative stress, inflammatory response, ECM degeneration, senescence and apoptosis are involoved in the occurrence of intervertebral disc degeneration (IDD). Mammalian target of rapamycin (mTOR) is an evolutionarily conserved serine/threonine kinase that plays a vital role in IDD progression. Recent studies have shown that mTOR signalling is involved in the regulation of autophagy, oxidative stress, inflammatory responses, ECM homeostasis, cellular senescence, and apoptosis in IVD cells. We reviewed the mechanism of mTOR signalling in the pathogenesis of IDD to provide innovative ideas for future research and IDD treatment.

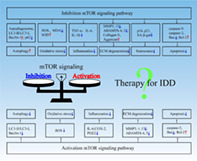

## 1 INTRODUCTION

Low back pain (LBP) is a common symptom of degenerative diseases of the musculoskeletal system, posing a serious medical and social problem worldwide. According to statistics, approximately 80% of the general population experiences LBP at least once during their lifetime, resulting in reduced patient quality of life and a heavy economic burden on society and patients.^1^, ^2^ According to a statistical analysis of the 2019 Global Burden of Disease Study, LBP increased disability‐adjusted life‐years by 46.9% from 1990 to 2019.^3^ Intervertebral disc degeneration (IDD) is an important pathological basis for a variety of degenerative spinal diseases, including lumbar intervertebral disc herniation (LDH), spondylolisthesis, and spinal stenosis, and is an important cause of chronic LBP.^4^, ^5^ However, the specific pathogenesis of IDD remains poorly understood. Currently, conservative treatment and surgical intervention can only relieve the clinical symptoms of IDD but fail to prevent or delay disease progression at the etiological level. Therefore, further exploration of IDD‐related molecular mechanisms would provide new strategies for LBP intervention.

The intervertebral disc (IVD) is a complex fibrocartilaginous tissue that is the most important functional part of the spine, located between upper and lower vertebrae, and plays an important role in carrying weight and buffering compressive loads.^6^, ^7^ Healthy IVDs are mainly composed of the central gelatinous nucleus pulposus (NP), inner and outer annulus fibrosus (AF) surrounding the NP, and cartilage endplate (CEP) above and below the NP and AF, forming a relatively closed environment.^8^, ^9^ The NP is primarily rich in type II collagen fibres, elastin fibres, and proteoglycans and is essential for spinal multiaxial flexibility and counteracting axial mechanical loads.^10^, ^11^ The AF consists of a series of concentric circular lamellar structures (type I and type II collagen fibres) divided into the inner and outer AFs. Compared with the inner AF, the outer AF contains more type I collagen fibres, which afford strong resistance to tensile load and prevent the NP from protruding outwards.^12^, ^13^ CEP is a thin hyaline cartilage that prevents the NP tissue from projecting into the vertebral body. Additionally, given that the IVD is an avascular tissue, NP and inner AF exchange nutrients and metabolic wastes by CEP diffusion, thereby maintaining normal IVD structure and function.^14^ Accumulated evidence suggests that inflammatory response, oxidative stress, autophagy, abnormal mechanical stress, nutritional deficiencies, and genetics can lead to reduced ECM synthesis and secretion by IVD cells, ultimately resulting in structural and functional dysfunction of the IVD.^15^, ^16^, ^17^

Mammalian target of rapamycin (mTOR) is an evolutionarily conserved serine/threonine kinase involved in the regulation of protein synthesis, cellular senescence, autophagy, apoptosis, and immunity.^18^ Several studies have confirmed that the mTOR signalling pathway plays an important role in various degenerative diseases, including osteoarthritis, diabetes, atherosclerosis, and Parkinson's disease.^19^, ^20^, ^21^, ^22^ Liu et al.^23^ used the LDH model to induce radicular pain in rats. Intraperitoneal injection of AMP‐activated protein kinase (AMPK) activators can activate the AMPK signalling pathway in dorsal root ganglion neurones, inhibit mTOR signalling, and alleviate LDH‐induced radicular pain, suggesting that inhibition of mTOR signalling can alleviate radicular pain caused by IVD protrusion‐mediated compression. Recent studies have suggested that mTOR signalling is critical for maintaining IVD homeostasis.^24^, ^25^ Based on existing literature, we focused on the mTOR signalling pathway and its multiple biological functions in IVD cells to comprehensively clarify the role of the mTOR signalling pathway in IDD.

## 2 STRUCTURE AND FUNCTION OF MTOR SIGNALLING PATHWAY

**FIGURE 1:**
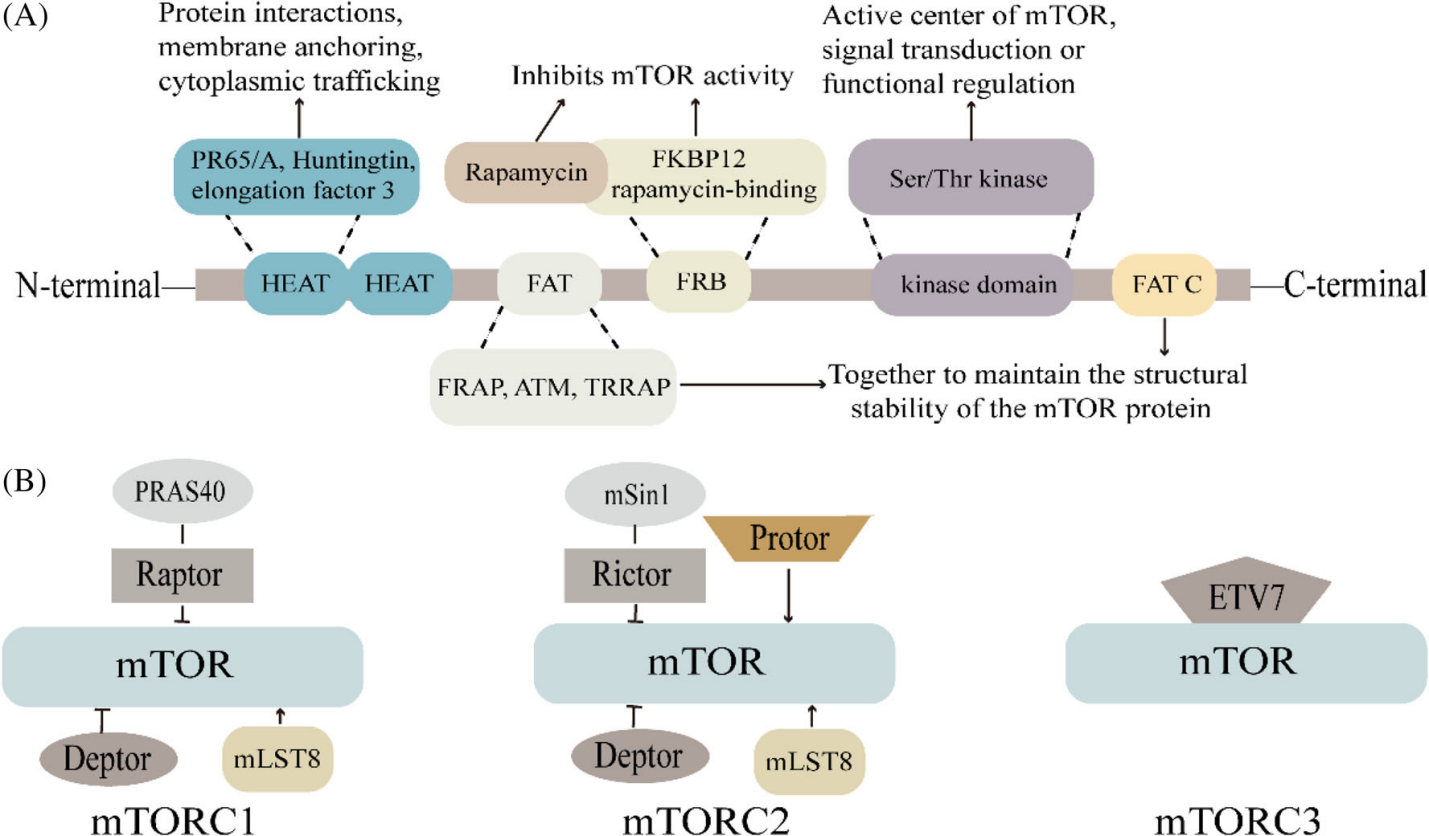
(A) Schematic diagram of the structure of mTOR and the functions of its components. (B) Schematic structure of mTORC1, mTORC2 and mTORC3. mTOR, mammalian target of rapamycin

mTOR is an atypical serine/threonine kinase belonging to the phosphatidylinositol kinase‐related kinase (PIKK) family.^26^ The mTOR protein is composed of five domains, each of which has a different function, including two groups of N‐terminal HEAT repeat (PR65/A subunit of protein phosphatase 2A, Huntingtin, elongation factor 3) domains, potentially involved in protein interactions, membrane anchoring, and cytoplasmic trafficking.^27^ The FAT (FRAP, ATM, TRRAP) domain occurs downstream of the HEAT repeat domain. In addition, the C‐terminal FAT C domain, which is similar to the FAT domain, maintains the structural stability of the mTOR protein.^28^ The FRB and Ser/Thr kinase domains are located between FAT and FATC domains. The FRB domain is the FKBP12‐rapamycin complex‐binding site of mTOR. Rapamycin binds to FKBP12 in the cytoplasm via the FRB domain, thereby inhibiting mTOR activity.^29^ The Ser/Thr kinase domain is the active centre of mTOR, which achieves signal transduction or functional regulation after activation.^30^ Following in‐depth investigations into mTOR protein, based on their structural and functional differences, mTOR can be divided into three distinct multi‐subunit protein complexes: mTOR complex 1 (mTORC1), mTOR complex 2 (mTORC2), and a putative mTOR complex 3 (mTORC3).^31^, ^32^ mTORC1 is composed of the catalytic subunit mTOR, a regulatory‐related protein of mTOR (Raptor), a proline‐rich Akt substrate (PRAS40), a DEP domain‐containing mTOR‐interacting protein (Deptor), and mammalian lethal protein SEC13 protein 8 (mLST8).^33^ In addition to the same three subunit catalytic subunits, mTOR, Deptor, and mLST8 in mTORC1, mTORC2 comprises three other subunits, including rapamycin‐insensitive companion of mTOR (Rictor), mitogen‐activated protein kinase‐related protein 1 (mSin1), and Protor.^34^ In mTORC1, Raptor acts as a bridge to PRAS40, whereas PRAS40 and mLST8 bind to mTOR as negative and positive regulatory subunits, respectively. In mTORC2, mSin1 localizes to Rictor, which, in turn, binds to mTOR and inhibits its activity. Protor is responsible for assisting in complex assembly, while mLST8 and Deptor function similarly to mTORC1.^29^ However, few reports are available on mTORC3. Current studies have revealed that mTOR3 is insensitive to rapamycin and has been shown to have tumourigenic effects; it is composed of ETV7, mTOR, and other undefined components, lacking Raptor or Rictor^35^ (Figure 1).

## 3 SIGNAL TRANSDUCTION OF MTOR SIGNALLING PATHWAY

The mTOR signalling pathway plays a critical role in regulating cell growth and metabolism in eukaryotic cells by modulating transcription, translation, lipid synthesis, autophagy, and lysosomal biosynthesis.^36^

Tuberous sclerosis complex (TSC1/TSC2) is a key negative regulator of mTORC1 activity.^37^ On exposure of cells to hypoxia, growth factors, energy stress, stress, and amino acids, mTORC1 is activated or inhibited through different pathways and participates in various biological processes. Downstream effectors of mTORC1, including eIF‐4E‐binding protein (4E‐BP1), sterol response element‐binding protein (SREBP), hypoxia‐inducible factor 1α (HIF‐1α), transcription factor EB (TFEB), and activating transcription factor 4 (ATF4), can regulate mRNA translation, lipid synthesis, glucose metabolism, lysosomal biogenesis, and nucleotide metabolism.^38^ The regulation of mRNA translation by mTORC1 is primarily mediated via the activation of ribosomal protein S6 kinase 1 (p70S6K) by 4E‐BP1. In addition, mTORC1 plays an important role in the regulation of autophagy. The ULK1‐Atg13 complex is conducive to the formation of autophagosomes, whereas mTORC1 phosphorylates ULK1 to facilitate complex formation with Atg13, thereby mediating the regulation of autophagy.^39^ Amino acids induce mTORC1 activation through small GTPases of the Rag family to coordinate nutrient requirements for cell growth.^40^ Growth factors then activate the PI3K‐Akt axis via corresponding receptors, and the activated Akt can inhibit complex TSC1/2 formation, thereby activating mTORC1.^32^ In addition, tumour necrosis factor‐alpha (TNF‐α) is phosphorylated by its downstream kinase IKKβ, which also leads to the activation of mTORC1 by inhibiting the formation of the TSC1/TSC2 complex.^41^ AMPK is a sensor of cellular energy levels, and mTORC1 signalling can also sense intracellular energy changes, suggesting a potential regulatory relationship. Studies have found that hypoxia and stress stimulation can activate the AMPK signalling pathway and phosphorylate Raptor, which reduces mTORC1 activity through allosteric inhibition, thereby regulating cell energy metabolism and promoting cell survival.^42^

**FIGURE 2:**
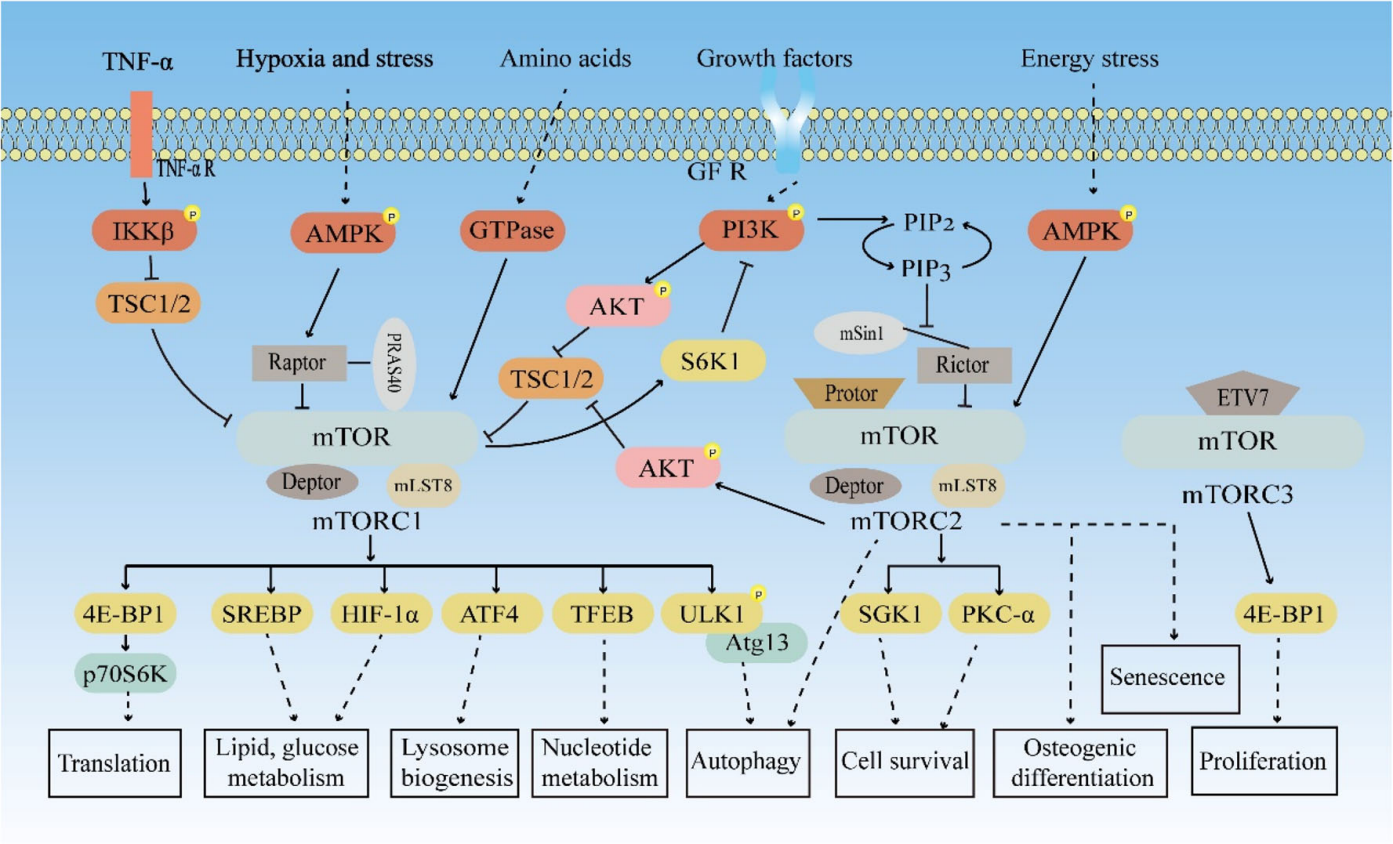
The mTOR‐mediated signalling pathway. Growth factors, hypoxia and stress, amino acids, energy stress, and TNF‐α activate mTORC1 by stimulating different signalling axes involved in regulating protein translation, lipid and sugar metabolism, lysosomal biogenesis, nucleotide metabolism, and autophagy. The activation of mTORC2 is involved in autophagy, cell survival, osteogenic differentiation, and senescence. The mTORC3 participates in cell proliferation. mTOR, mammalian target of rapamycin; TNF‐α, tumour necrosis factor‐alpha

However, compared with mTORC1, the molecular regulation of mTORC2 and downstream signalling was relatively reduced. mTORC2 has been shown to play important roles in regulating endocytosis, sphingolipid biosynthesis, cell survival, and actin cytoskeleton reorganization.^43^ During growth factor stimulation, PI3K phosphorylates PI(4,5)P2 to generate PI(3,4,5)P3 and subsequently binds to mSIN1 to relieve mTORC2 inhibition via mSin1, further leading to mTORC2 activation, which, in turn, phosphorylates Akt, which is involved in the regulation of biological processes.^44^ In response to energy stress, the AMPK signalling pathway can directly activate mTORC2 to enhance cell survival.^45^, ^46^ In addition, mTORC2 can promote cell survival through downstream genes, serum and glucocorticoid‐induced protein kinase 1 (SGK1) and protein kinase C‐α (PKC‐α).^47^ Studies have shown that mTORC1 can downregulate PI3K signalling via S6K1, resulting in mTORC2 inactivation, suggesting a potential feedback control loop between mTORC1 and mTORC2.^44^ Recent studies have shown that mTORC2 is also involved in regulating autophagy, cell senescence, and induction of osteogenic differentiation.^48^, ^49^ In addition, studies have shown that mTORC3 affects the proliferation of tumour cells through 4E‐BP1^35^; however, the underlying regulatory mechanism remains elusive, warranting in‐depth future investigations (Figure 2).

## 4 MECHANISMS UNDERLYING MTOR SIGNALLING IN IVD CELLS

**FIGURE 3:**
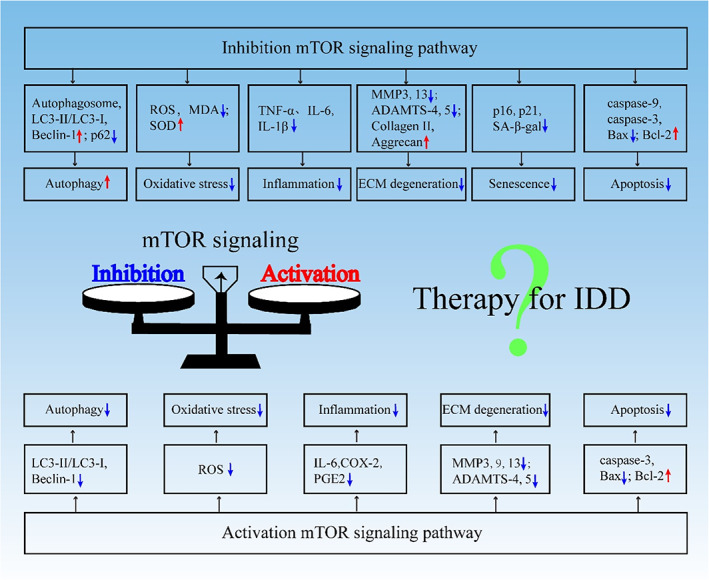
Challenges of mTOR‐based treatment of IDD. Autophagy, oxidative stress, inflammation, ECM homeostasis, senescence, and apoptosis mediated by mTOR signalling can markedly influence IVD cell fate and IDD pathophysiology. Inhibiting or activating mTOR signalling can affect the above‐mentioned pathophysiological processes and is beneficial to delay the progression of IDD. Therefore, the function of targeting mTOR signalling in IDD requires further clarification. IDD, intervertebral disc degeneration; IVD, intervertebral disc; mTOR, mammalian target of rapamycin

IDD is a complex degenerative disease of the musculoskeletal system mediated by multiple pathological processes. Studies have shown that autophagy, oxidative stress, inflammatory responses, imbalances in ECM synthesis and catabolism, and genetic factors can crucially contribute to IDD.^50^ Numerous studies have shown that the mTOR signalling pathway plays an important role in regulating cell growth and metabolism. Recent studies have found that human IVD NP tissue exhibit the expression of molecules related to mTOR signalling; however, owing to insufficient tissue sample size, the correlation between its expression level and grade of IDD degeneration needs to be further explored.^51^, ^52^ NP cells treated with high oxygen tension showed increased levels of intracellular reactive oxygen species (ROS) and ECM degradation.^53^ In addition, compared with the normal group, high oxygen tension leads to abnormal expression of various genes in NP cells, and the Kyoto Encyclopedia of Genes and Genome pathway analysis showed that the response of NP cells to high oxygen tension involves multiple pathways, including the mTOR signalling pathway.^53^ In IDD, compared with mTORC2 inhibition, mTORC1 inhibition enhanced autophagy in NP cells, thereby reducing NP cell apoptosis, senescence, and ECM degradation, suggesting that mTORC1 may play a key role in IDD progression.^51^, ^52^ Therefore, the mTOR signalling pathway participates in the regulation of autophagy, oxidative stress, inflammation, apoptosis, and ECM homeostasis in IDD (Figure 3).

### 4.1 mTOR signalling and autophagy in NP

Autophagy is the catabolic mechanism employed by eukaryotic cells to maintain nutrient homeostasis, which captures and degrades misfolded proteins and damaged or aged organelles in lysosomes, while recycling intracellular components to maintain intracellular homeostasis.^54^, ^55^ Recent studies have suggested that autophagy disorders in IVD cells may be an important factor in IDD.^56^, ^57^ Compared with normal IVD, the IVD of IDD exhibited fewer autophagosomes, Beclin‐1, and a lower ratio of LC3‐II to LC3‐I.^58^ Interestingly, Gruberde et al. found increased expression of autophagy‐related genes (Beclin‐1, ATG8, and ATG12) in the IVD of IDD.^59^ Furthermore, studies have found that appropriate autophagy activity is beneficial to the survival of NP cells during serum deprivation, while excessive autophagy leads to NP cell death.^60^ Therefore, body‐induced autophagy increases in the early stages of in vitro degeneration, which may provide a protective mechanism for in vitro cells. Under the continuous action of various unfavourable factors, it can lead to excessive activation of autophagy, engulfing normal organelles or degrading normal proteins, thereby increasing the potential for cell death and accelerating the IDD process.

The mTOR signalling pathway and autophagy have attracted increasing attention from researchers.^61^ Studies have shown that the mTOR signalling pathway can regulate autophagy in chondrocytes and has a beneficial effect on rat osteoarthritis (OA).^62^ Tu et al.^63^ found that the expression of sestrins was lower in NP tissues of patients with IDD. Overexpression of sestrins could inhibit mTOR activity in NP cells, resulting in an increased LC3‐II/I ratio and decreased p62 expression, reducing endoplasmic stress‐induced apoptosis and ECM degeneration. However, decorin, increased in NP tissues of patients with IDD, reportedly suppresses mTOR phosphorylation by inhibiting the PI3K/AKT signalling pathway, thereby promoting autophagy and reducing apoptosis in rat NP cells.^64^ We previously reported that bromodomain‐containing protein 4 (BRD4) expression was elevated in NP tissues of patients with IDD. Silencing of BRD4 can activate the AMPK pathway in human NP cells, inhibit mTOR activity, and promote the phosphorylation of ULK1, increasing the LC3‐II/LC3‐I ratio and Beclin‐1 levels and decreasing the level of P62.^65^ Immunofluorescence (LC3) and transmission electron microscopy (autophagosome formation) further confirmed that silencing BRD4 promoted autophagy and reduced apoptosis and senescence.^65^ In short, the above‐listed studies have shown that inhibition of the mTOR signalling pathway promotes autophagy, which is beneficial for the survival of NP cells and the maintenance of physiological functions. In addition, various natural compounds can inhibit mTOR activity, which provides a new approach for the development of drugs targeting mTOR to treat IDD (Table 1).

**TABLE 1:** Drugs or genes that positively or negatively regulate mTOR activation in IDD

Name	Cell	Stimulation	mTOR activity	Function	References
Curcumin	NPc	H_2_O_2_	Downregulated	Promote autophagy, inhibit oxidative stress, apoptosis, senescence, ECM degeneration	73
Quercetin	NPc	THBP	Downregulated	Promote autophagy, inhibit apoptosis, ECM degeneration	92
Apigenin	NPc	THBP	Downregulated	Promote autophagy, inhibit apoptosis, senescence, ECM degeneration	96
Moracin	NPc	LPS	Downregulated	Promote autophagy, inhibit inflammation, ECM degeneration	80
Glucosamine	NPc	IL‐1β/ H_2_O_2_	Downregulated	Promote autophagy, inhibit apoptosis, senescence, ECM degeneration	91
PTH	NPc	dexamethasone	Downregulated	Promote autophagy, inhibit senescence	97
Sestrins	NPc	2‐deoxyglucose	Downregulated	Promote autophagy, inhibit apoptosis, ECM degeneration	63
Decorin	NPc	IL‐1β	Downregulated	Promote autophagy, inhibit apoptosis, ECM degeneration	64
BRD4	NPc	IL‐1β	Downregulated	Promote autophagy, inhibit apoptosis, senescence, ECM degeneration	65
TNFAIP3	NPc	LPS	Downregulated	Promote autophagy, inhibit inflammation, ECM degeneration	24
p65	NPc	LPS	Downregulated	Promote autophagy, inhibit inflammation	81
IAPP	NPc	Overexpression IAPP	Downregulated	Promote autophagy, inhibit apoptosis, ECM degeneration	90
OP‐1	NPc	hypertonic	Upregulated	Inhibit apoptosis	102
Liraglutide	NPc	high glucose	Upregulated	Inhibit apoptosis	103
Resveratrol	NPc	IL‐1β	Upregulated	Inhibit apoptosis	104
17β‐oestradiol	NPc	IL‐1β	Upregulated	Inhibit apoptosis	105
BM‐MSCs	AFc	IL‐1β	Upregulated	Inhibit apoptosis, inflammation	110
TGF‐β1	AFc	serum deprivation	Upregulated	Promote autophagy, inhibit apoptosis	111
1,25(OH)2D3	AFc	H_2_O_2_	Downregulated	Promote autophagy, improve mitochondrial function, inhibit apoptosis	108
Rapamycin	AFSCs	bleomycin	Downregulated	Inhibit inflammation, senescence	109
Bafilomycin A1	CEPc	H_2_O_2_	Downregulated	Promote autophagy, inhibit apoptosis	112

Abbreviations: AFc, annulus fibrosus cells; AFSCs, annulus fibrosus stem cells; CEPc, cartilage endplate cells; ECM, extracellular matrix; mTOR, mammalian target of rapamycin; NPc, nucleus pulposus cells.

### 4.2 mTOR signalling and oxidative stress in NP

Oxidative stress is defined as an imbalance between ROS production in the body and endogenous antioxidant defence mechanisms, resulting in disrupted redox signalling and related molecular damage.^66^ Mitochondria are major sites of ROS production. Under the stimulation by unfavourable factors, excessive ROS production by cells can cause protein and lipid peroxidation and DNA damage, leading to cellular dysfunction or irreversible cell damage and death.^67^ Chen et al.^68^ reported that t‐butyl hydroperoxide (TBHP)‐induced oxidative stress promoted senescence and apoptosis in NP cells in vitro. In addition, ROS can lead to increased CEP apoptosis and promote CEP calcification via the MAPK/NF‐κB pathway.^69^ Therefore, oxidative stress may be an important contributing factor to the acceleration of IDD.

The mTOR signalling pathway has been confirmed to be closely related to the regulation of oxidative stress. The neurological function and oxidative stress state of patients with acute stroke were improved through the mTOR signalling pathway.^70^ Dai et al.^71^ reported that mTOR‐mediated autophagy could alleviate oxidative stress damage in chondrocytes and inhibit the progression. According to Wu et al.,^72^ compared with the normoxia group, pramlintide showed superior inhibition of ROS production and reduced apoptosis and ECM degeneration in NP cells under hypoxic conditions, which activated the AKT‐AMPK/mTOR signalling pathway. Similarly, Chen et al.^60^ found that hypoxia can inhibit the AMPK/mTOR signalling pathway, thereby reducing excessive mitochondrial ROS production and apoptosis in NP cells under serum deprivation. The above studies demonstrated that activating the mTOR signalling pathway under hypoxic conditions could effectively alleviate ROS‐induced damage to NP cells. However, studies have shown that mTOR inhibition can also reduce ROS levels in NP cells. Kang et al.^73^ demonstrated that curcumin increased the activity of antioxidants (superoxide dismutase [SOD]) and enhanced NP cell autophagy by reducing levels of ROS and malondialdehyde in TBHP‐treated human NP cells by activating the AMPK/mTOR signalling pathway. The difference between these two results may be explained by the existence of the NP in a hypoxic environment. NP cells downregulate their cellular metabolic rate to a new low metabolic homeostasis by balancing ATP demand and ATP supply pathways, of which activation of the mTOR signalling pathway may be a potential regulatory mechanism. Interestingly, upon the continuous action of unfavourable factors, NP cells may increase autophagic flux to remove intracellular ROS and reduce cell damage by inhibiting mTOR activity. This suggests that autophagy, mediated by the mTOR signalling pathway, plays a crucial role in regulating oxidative stress under unfavourable conditions, but the underlying mechanism remains unclear. Therefore, it is necessary to comprehensively explore the specific mechanism of mTOR signalling in the regulation of oxidative stress (Table 1).

### 4.3 mTOR signalling and inflammation in NP

Inflammation is an important driver accelerating the pathogenesis of IDD. IVD regression is often accompanied by the infiltration of mast cells, macrophages, and neutrophils, which secrete various inflammatory mediators, including TNF‐α, interleukin (IL)‐1α/β, IL‐6, IL‐8, IL‐17, and prostaglandin E2 (PGE2).^74^ As TNF‐α and IL‐1β exhibit potent pro‐inflammatory activities, they have been extensively studied in IDD. TNF‐α and IL‐1β significantly increased the expression of pro‐inflammatory mediators (IL‐6, IL‐8, inducible nitric oxide synthase [iNOS], and prostaglandin‐endoperoxide synthase 2 [PTGS2]) in NP cells in vitro and aggravated inflammatory damage in NP cells.^75^ In addition, TNF‐α and IL‐1β were shown to upregulate thrombospondin‐motif disintegrins and metalloproteinases (ADAMTS‐4 and ‐5) and matrix metalloproteinases (MMP‐1, ‐2, ‐3, ‐13, ‐14) in IVD cells, thereby promoting ECM degradation and accelerating IVD degeneration.^76^

It has been reported that mTOR signalling is also involved in the regulation of inflammatory responses.^77^ Xue et al.^78^ reported that the mTOR signalling pathway is involved in the regulation of autophagy and inflammatory responses in chondrocytes. TNF‐α‐inducible protein 3 (TNFAIP3) is a ubiquitin‐modifying enzyme primarily involved in the regulation of mTOR signalling pathway.^79^ Chen et al.^24^ found that TNFAIP3 inhibited the mTOR signalling pathway to promote autophagy, reduce the expression of TNF‐α and IL‐1β, and inhibit ECM degeneration in human NP cells. While pretreatment with mTOR inhibitor (Torin1), TNFAIP3‐induced autophagy was attenuated, and inflammation was exacerbated, suggesting that activation of autophagy reduced inflammation.^24^ Moracin inhibited the expression of TNF‐α, IL‐6, and IL‐1β in LPS‐induced rat NP cells and promoted autophagy by suppressing the PI3K/AKT/mTOR signalling pathway.^80^ In addition, Yi et al.^81^ reported that p65‐siRNA transfection inhibited the NF‐κB pathway, significantly reduced p‐AKT and p‐mTOR expression, promoted autophagy, and reduced LPS‐induction inflammation in human NP cells. Autophagy mediated by the mTOR signalling pathway may play a key role in regulating NP cell inflammation. Thus, further research will provide novel insights into the inflammatory regulation of mTOR in NP cells (Table 1).

### 4.4 mTOR signalling and ECM degradation in NP

ECM synthesized and secreted by IVD cells plays an important role in maintaining the IVD height and buffering the mechanical load from the spine.^82^ ECM is composed of collagen types I and II, aggrecan, elastic fibres, hyaluronic acid, chondroitin sulphate, water, and glycoproteins.^83^ Numerous studies have demonstrated that excessive disruption of the ECM, particularly insufficient synthesis or increased degradation of type II collagen and aggrecan, leads to structural and functional impairment of the IVD, thereby accelerating the progression of IDD.^84^ ADAMTS and MMPs are major enzymes responsible for ECM degradation.^85^ Compared with normal IVD, collagen type II and aggrecan expression were reduced in the IVD of IDD.^86^, ^87^ However, most MMPs (MMP‐1, ‐2, ‐3, ‐8, ‐9, ‐10, ‐12, ‐13, ‐14) and ADAMTS (ADAMTS‐4, ‐5) showed increased expression in degenerative IVD tissues.^85^, ^88^ In OA, mTOR signalling was involved in regulating MMPs and ADAMTS expression, thereby reducing ECM degradation.^89^ Wu et al.^90^ demonstrated that, compared with the knockout group, overexpression of Islet amyloid polypeptide (IAPP) promoted autophagy in NP cells via PI3K/Akt/mTOR and JNK signalling pathways, significantly reduced the expression of TNF‐α, IL‐1, MMP3, MMP9, MMP13, ADAMTS‐4, and ADAMTS‐5, promoted the expression of collagen II and aggrecan, and reduced apoptosis. Glucosamine inhibited mTOR activity to promote autophagy in NP cells, decreased the expression of matrix‐degrading enzymes (MMP3, MMP13, and ADAMTS‐5), and increased the expression of collagen II and aggrecan; while the autophagy inhibitor (3‐MA) attenuated these protective effects.^91^ Zhang et al.^92^ suggested that quercetin inhibited the p38MAPK signalling pathway to inhibit mTOR activation in TBHP‐induced rat NP cells, inhibiting ECM degradation and apoptosis. In addition, activation of the AMPK/mTOR signalling pathway promoted autophagy in NP cells, thereby inhibiting the expression of MMP3 and ADAMTS‐4 in human NP cells induced by IL‐1β and increasing the expression of collagen II and aggrecan.^65^, ^73^ Accordingly, mTOR‐mediated autophagy appears to play an important role in ECM metabolism; however, its potential regulatory mechanism remains poorly understood, thus warranting further in‐depth investigations (Table 1).

### 4.5 mTOR signalling and senescence in NP

Senescence typically refers to the gradual decline in cell proliferation and differentiation ability or irreversible cell cycle arrest caused by various external stimuli, resulting in physiological dysfunction of cells.^93^ Cellular senescence is divided into replicative and stress‐induced premature senescence. Replicative senescence is caused by telomere shortening, which results from continuous cell replication. Cellular senescence induced by exogenous stress, such as inflammatory response, DNA damage, oxidative stress, nutrient deprivation, and mitochondrial dysfunction, is called stress‐induced premature senescence.^94^ Cellular senescence is also an important factor leading to IVD regression. Senescence‐associated secretory phenotypes (SASPs) are secreted by senescent cells, including pro‐inflammatory cytokines, growth factors, cytokines, matrix‐degrading proteases, and other bioactive factors, disrupting the balance between IVD ECM synthesis and catabolism.^16^ In addition, SASP can cause changes in the cellular microenvironment in an autocrine or paracrine manner, further accelerating the senescence of self or neighbouring cells.^95^ Therefore, inhibition of cellular senescence might afford a new strategy for delaying IDD. Silencing BRD4 reduced the expression of p16 and p21 in IL‐1β‐induced human NP cells and the positive rate of senescence‐associated β‐galactosidase cells by activating the AMPK/mTOR pathway.^65^ Additionally, the downstream TFEB gene of the AMPK/mTOR pathway may play an important role in regulating NP cell senescence. Apigenin activates the AMPK/mTOR pathway and promotes the entry of the downstream TFEB gene into the nucleus, promoting autophagy and inhibiting NP cell senescence. Following transfection with siRNA‐TFEB, apigenin failed to inhibit the senescence of NP cells induced by THBP.^96^ What's more, Wang et al.^97^ reported that parathyroid hormone 1–34 (PTH) could inhibit the mTOR signalling pathway, promote autophagy in rat NP cells, and suppress NP cell senescence; transfection of siRNA‐ATG5 inhibited autophagy, and the protective effect of PTH was abolished. These studies suggest that inhibition of the mTOR pathway might suppress NP cell senescence. Moreover, inhibition of mTOR signalling restores autophagy, thereby degrading obsolete or damaged cellular proteins and organelles and salvaging them for “spare macromolecular parts” to provide raw materials for proliferating cells. In summary, the mTOR signalling pathway is crucial for regulating cellular senescence (Table 1).

### 4.6 mTOR signalling and apoptosis in NP

Apoptosis is programmed cell death regulated by multiple signal transduction pathways, characterized by chromosome condensation, cell shrinkage, DNA degradation, and apoptotic body formation.^98^ A high apoptosis rate was observed in IVD‐degenerated tissue specimens.^99^ Increased apoptosis leads to a decreased number of cells within the IVD, which, in turn, disrupts tissue homeostasis and plays an important role in IDD pathogenesis.^100^ Therefore, inhibition of apoptosis could be a potentially attractive therapeutic strategy for IDD. mTOR signalling also plays an important role in the regulation of apoptosis. In IDD, compression can activate the JNK signalling pathway and inhibit the Akt/mTOR signalling pathway to promote autophagy in NP cells and reduce apoptosis.^101^ Xie et al.^96^ reported that the downstream TFEB gene of the AMPK/mTOR pathway plays an important role in the anti‐apoptotic process of apigenin. When transfected with siRNA‐TFEB, apigenin did not inhibit THBP‐induced apoptosis of NP cells. The above studies revealed that inhibition of the mTOR signalling pathway could inhibit the apoptosis of NP cells. Therefore, targeting the mTOR signalling pathway for inhibition of apoptosis may be a promising approach for treating IDD. However, some studies have confirmed that the activation of the mTOR signalling pathway can also inhibit the apoptosis of NP cells. Yang et al.^102^ reported that osteogenic protein 1(OP‐1) activates the PI3K/Akt/mTOR signalling pathway and inhibits rat NP cell apoptosis induced by hypertonic culture; while pretreatment with PI3KAkt inhibitor (LY294002) suppressed mTOR activation, and apoptosis of rat NP cells was increased. In addition, liraglutide activated the PI3K/Akt/mTOR signalling pathway and inhibited high glucose‐induced apoptosis of NP cells.^103^ Similarly, both resveratrol and 17β‐oestradiol activated mTOR signalling and reduced the expression of caspase‐3 induced by IL‐1β in NP cells, thereby inhibiting apoptosis; treatment with an mTOR inhibitor (rapamycin) abolished their apoptosis‐inhibiting effect.^104^, ^105^ Collectively, the mechanism underlying mTOR signalling in the regulation of apoptosis remains unclear, but the activation of autophagy may inhibit excessive apoptosis. Therefore, it is necessary to further investigate the potential regulatory relationship between mTOR, autophagy, and apoptosis to provide a basis for developing biological therapies for IDD (Table 1).

### 4.7 mTOR signalling in AF and CEP

AF plays a critical role in IVD homeostasis. AF consists of a series of concentric circular lamellae with fibres in adjacent lamellae, approximately ±60° from the orientation of the spinal axis, which helps AF tissue support multidirectional loading during normal activity.^106^ When the spine is subjected to axial compression, the tightly packed annulus fibrosus absorbs pressure from the NP to the AF wall.^107^ Therefore, AF degeneration accelerates the progression of IDD. Recent studies have shown that the mTOR signalling pathway participates in the regulation of AF cell homeostasis. 1,25(OH)2D3 binds via its vitamin D receptor and activates autophagy by inhibiting the mTOR/p70S6K signalling pathway, effectively decreasing the level of H_2_O_2_‐induced ROS in AF cells and increasing mitochondrial ATP content to improve mitochondrial function and inhibit AF cell apoptosis.^108^ Rapamycin, an mTOR inhibitor, significantly inhibited bleomycin‐induced expression of inflammatory factors (IL‐1β, IL‐6, and TNF‐α) and senescence in rabbit AF stem cells (AFSCs).^109^ These findings suggest that the inhibition of mTOR signalling reduces inflammation, apoptosis, and senescence. However, other studies have reported contradictory results. Bone marrow mesenchymal stem cell‐derived exosomes (BM‐MSCs) can activate the PI3K/AKT signalling pathway, thereby activating mTOR, inhibiting IL‐1β‐induced apoptosis, and the expression of inflammatory factors (IL‐6, COX‐2, and PGE2) induced by IL‐1β in AF cells.^110^ In addition, the exogenous addition of TGF‐β1 significantly upregulated the activities of AKT/mTOR signalling and downregulated the expression of autophagy proteins Beclin‐1 and LC3 II/I in cells under serum deprivation, thereby inhibiting apoptosis in AF cells.^111^

Given that IVD is an avascular tissue, most nutrients and metabolic wastes are mainly exchanged through CEP; accordingly, CEP plays an important role in the nutrient supply to the IVD. Therefore, CEP degeneration can also accelerate the progression of IDD. In CEP cells, H_2_O_2_ stimulation resulted in a time‐dependent increase in cell apoptosis, whereas the expression of p‐mTOR and p‐p70S6K1 and the ratio of autophagy‐related genes LC3‐II/LC3‐I was first increased, followed by a gradual decrease. Following treatment with an autophagy inhibitor (bafilomycin A1), H_2_O_2_‐induced apoptosis of CEP cells was further aggravated.^112^ This observation suggests that cell stimulation by adverse factors may temporarily activate autophagy to play a protective role; cells are more susceptible to damage and death when autophagy is completely suppressed. To date, few studies have investigated the role of mTOR signalling in AF and CEP; however, given its involvement in the multiple pathophysiological mechanisms regulating IDD, further studies are required. Among these, mTOR‐mediated autophagy plays a key role in cell survival. Moderate autophagy confers a protective effect on cells, whereas inhibition of autophagy or excessive autophagy can lead to accelerated cell death. Additional research on its potential mechanism is needed, which would be of considerable significance for IDD therapy (Table 1).

## 5 MTOR SIGNALLING AND NONCODING RNAS (NCRNAS) IN IDD

Previous studies have shown that approximately 70% of patients with IDD exhibit genetic variants, suggesting that genetics may be a key factor in the pathogenesis of IDD.^113^ With the development of sequencing technology, it was reported that ncRNAs account for up to 98% of the entire human genome, thereby indicating that ncRNAs play an important role in biological regulation. There are three types of ncRNAs: microRNAs (miRNAs), long ncRNAs (lncRNAs), and circular RNA (circRNAs). Growing evidence suggests that ncRNAs are involved in the development of IDD.^114^, ^115^ Herein, we summarized ncRNAs associated with mTOR signalling in IVD cells (Table 2).

**TABLE 2:** Noncoding RNAs involved in mTOR signalling in IDD

Non‐coding RNA(s)	Expression level in IDD	Target Gene(s)	Cells	mTOR activity	Functional role	References
miRNA‐21	Upregulated	PTEN	NPc	Upregulated	Inhibits autophagy in NP cells and promotes ECM degradation	116
miR‐654‐5p	Upregulated	ATG7	NPc	Upregulated	Inhibits autophagy in NP cells and promotes ECM degradation	117
miRNA‐143‐5p	Upregulated	eEF2	NPc	Upregulated	Inhibits NP cell proliferation, promotes apoptosis and senescence	118
miR‐19b‐3p	Downregulated	PTEN	NPc	Upregulated	Inhibition of apoptosis and ECM degradation in NP cells	25
miRNA‐32‐5p	Downregulated	PTEN	NPc	Upregulated	Promotes NP cell proliferation and inhibits apoptosis	119
lncRNA HOTAIR	Upregulated		NPc	Downregulated	Promotes autophagy in NP cells, accelerates apoptosis and senescence	120

Abbreviations: ECM, extracellular matrix; IDD, intervertebral disc degeneration; IVD, intervertebral disc; mTOR, mammalian target of rapamycin; NP, nucleus pulposus.

miRNAs are a group of small, evolutionarily conserved non‐coding RNAs of approximately 20–25 nucleotides in length that regulate messenger RNA (mRNA) translation by binding to target genes through specific sequences, thereby blocking protein expression or inducing mRNA degradation.^121^ Increased expression of miRNA‐21 and miR‐654‐5p has been reported in IVD tissues. Wang et al.^116^ found that the expression of miRNA‐21 was increased in the IVD tissues of patients with IDD, and miRNA‐21 could upregulate the expression of MMP‐3 and MMP‐9 in human NP cells to promote ECM degradation and inhibit autophagy in NP cells. Mechanistically, overexpression of miRNA‐21 activates the PI3K/Akt signalling pathway by targeting PTEN, thereby activating mTOR, inhibiting autophagy in human NP cells, and promoting ECM degradation; however, overexpressed miRNA‐21‐induced ECM degradation was blocked in the presence of an mTOR inhibitor (sirolimus).^116^ Similarly, miR‐654‐5p targeting ATG7 activated the P13K/AKT/mTOR signalling pathway, inhibited autophagy, and promoted the expression of ECM‐degrading enzymes (MMP‐3, MMP‐9, and MMP‐13) in human NP cells, thereby inhibiting the expression of type II collagen, SOX9, and aggrecan.^117^ These findings suggest that the activation of mTOR signalling can inhibit autophagy in NP cells and promote ECM degradation. In addition, miRNA‐143‐5p was upregulated in the NP tissues of patients with IDD. miRNA‐143‐5p targeting the eEF2 gene inhibited the AMPK signalling pathway in human NP cells, leading to mTOR activation, inhibition of NP cell proliferation, and increased apoptosis and senescence.^118^ The findings of the above studies suggest that the activation of mTOR signalling may be an important factor contributing to the deceleration of IDD progression. Moreover, studies have shown that the activation of mTOR signalling could delay the progression of IDD. The expression of miR‐19b‐3p and miRNA‐32‐5p was decreased in the IVD tissues of patients with IDD, whereas PTEN expression was increased. Zhao et al.^25^ reported that overexpression of miR‐19b‐3p targeting PTEN and activation of PI3K/Akt/mTOR reduced apoptosis, as well as the expression of ECM‐degrading enzymes (MMP‐3, MMP‐9, MMP‐13, ADAMTS‐4, and ADAMTS‐5). Interestingly, ECM degradation was increased in the presence of torin1, an inhibitor of the mTOR signalling pathway.^25^ In addition, Zhan et al.^119^ found that transfection of miRNA‐32‐5p mimic targeted the PTEN gene, activated the PI3K/Akt signalling pathway, promoted mTOR activity, facilitated NP cell proliferation, and inhibited apoptosis (Table 2).

lncRNAs are a group of RNA transcripts longer than 200 nucleotides that do not encode proteins and play important functional roles in regulating the transcription and translation of metabolism‐related genes.^122^ Recent studies have shown that lncRNAs are closely associated with the occurrence of IDD.^123^ Zhan et al.^120^ found that the expression of lncRNA HOTAIR and autophagy‐related genes (Beclin‐1 and LC3‐II) was higher in the NP tissue of patients with IDD than in the normal group. The authors also confirmed that overexpression of lncRNA HOTAIR could promote autophagy in human NP cells by activating the AMPK/mTOR/ULK1 signalling pathway and accelerating the apoptosis and senescence of NP cells; in the presence of autophagy inhibitor 3‐MA, apoptosis and senescence induced by overexpression of lncRNA HOTAIR was inhibited.^120^ This suggests that excessive activation of autophagy further aggravates senescence and apoptosis of NP cells. In addition, several studies have confirmed that circRNAs are important factors involved in the occurrence and development of IDD.^124^ However, no study has reported the regulation of the mTOR signalling pathway by circRNAs. Therefore, further in‐depth investigations are needed to develop a promising biological therapy for restoring the expression of downregulated ncRNAs or silencing aberrantly upregulated ncRNAs to impact the process of IDD via the mTOR signalling pathway (Table 2).

## 6 CONCLUSION AND PROSPECTS

LBP is considered a serious medical and social problem worldwide, seriously affecting the quality of life of patients and imposing a heavy economic burden. IDD is an important factor leading to LBP. The process of IDD development is often accompanied by abnormal autophagy, participation of inflammatory mediators, increased oxidative stress, abnormal increase in apoptosis and ageing, and imbalance of ECM synthesis and catabolism. Recent studies have demonstrated the importance of mTOR signalling in the regulation of autophagy, inflammation, oxidative stress, apoptosis, senescence, and the metabolic processes of the ECM in IVD cells; however, the underlying mechanisms remain largely unknown. Therefore, targeting mTOR signalling may be a promising therapeutic approach for treating IDD. Despite our basic understanding of the relationship between the mTOR signalling pathway and IDD, several questions remain unanswered. (1) Most studies have confirmed that inhibiting the mTOR signalling pathway can promote autophagy, inhibit IVD inflammation, apoptosis, and senescence, and increase ECM synthesis. Inhibition of mTOR signalling promotes autophagy, which suppresses apoptosis, senescence, and ECM degeneration. Therefore, restoring autophagic flux is crucial for delaying IDD progression. However, current studies have revealed that excessive autophagy can accelerate cell death^60^, ^120^; therefore, regulating mTOR to maintain an appropriate level of autophagy and exert a protective effect on cells is urgently needed in IDD. Additionally, a few studies have shown that activation of the mTOR signalling pathway can also reduce cell apoptosis and inflammation‐induced damage. However, these studies have rarely investigated whether autophagy is involved in regulating apoptosis and inflammation, which may be the main reason for the divergent results. Therefore, there is an urgent need to further explore the specific mechanism of mTOR signalling in IDD, which brings both opportunities and challenges to its treatment. (2) The development of drugs targeting ncRNAs or key genes in diseases is a novel and promising therapeutic strategy. Therefore, further studies on the molecular mechanisms of ncRNAs and mTOR in IDD are needed. (3) Studies on mTOR and IDD have only been validated in vitro and in animal models, and clinically relevant data are lacking. Further translational research and clinical trials are required to evaluate the efficacy and safety of mTOR targeting in IDD therapy.

Recent studies have shown that mTOR is a key regulator of immune responses. mTOR signalling can play an important role by integrating various signals from the immune microenvironment and coordinating immune cell function and metabolism.^125^, ^126^ NP is an immune‐privileged organ that remains unrecognized by the immune system under normal physiological conditions. However, following IVD degeneration, nerves and blood vessels grow into internal AF and NP, and various immune cells (macrophages, T lymphocytes, glial cells) are recruited and react with NP to produce autoantibodies, thereby triggering an immune response and releasing cytokines to amplify inflammation, accelerating IDD progression.^127^, ^128^ However, mTOR signalling is yet to be implicated in the regulation of immune function in IDD, which might be a potential target for developing new therapies. Moreover, the mTOR signalling pathway is known to be involved in glycolysis, pyroptosis, and ferroptosis in various diseases.^129^, ^130^, ^131^ In IDD, glycolysis is the main pathway of energy metabolism in NP cells, and inhibition of glycolysis in NP cells can significantly impact their normal physiological functions.^132^ Inhibition of pyroptosis or ferroptosis was found to delay IDD progression.^133^, ^134^ Furthermore, the functions of the mTOR signalling pathway in glycolysis, pyroptosis, and ferroptosis are yet to be explored in IVDs, which might provide new ideas for assessing the mTOR pathway in IDD. In conclusion, a comprehensive understanding of the relationship between mTOR and IDD will provide a clear theoretical basis for the development of safe and effective mTOR pathway‐targeted drugs for IDD.

## AUTHOR CONTRIBUTIONS

Hai‐Wei Chen, Jian‐Wei Zhou, and Guang‐Zhi.Zhang contributed equally to this work and is listed as a co‐first author. All authors contributed to the revision and approved the submitted version.

## CONFLICT OF INTEREST

The authors declare no conflicts of interest.

## Data Availability

Data sharing not applicable to this article as no data sets were generated or analysed during the current study.
